# Quantifying Systemic Risk by Solutions of the Mean-Variance Risk Model

**DOI:** 10.1371/journal.pone.0158444

**Published:** 2016-06-28

**Authors:** Jan Jurczyk, Alexander Eckrot, Ingo Morgenstern

**Affiliations:** Department of Physics, University of Regensburg, Regensburg, Germany; East China University of Science and Technology, CHINA

## Abstract

The world is still recovering from the financial crisis peaking in September 2008. The triggering event was the bankruptcy of Lehman Brothers. To detect such turmoils, one can investigate the time-dependent behaviour of correlations between assets or indices. These cross-correlations have been connected to the systemic risks within markets by several studies in the aftermath of this crisis. We study 37 different US indices which cover almost all aspects of the US economy and show that monitoring an average investor’s behaviour can be used to quantify times of increased risk. In this paper the overall investing strategy is approximated by the ground-states of the mean-variance model along the efficient frontier bound to real world constraints. Changes in the behaviour of the average investor is utlilized as a early warning sign.

## Introduction

Systemic risk is a financial term used to describe the probability of a breakdown in an entire system [1, 2]. A typical example is a bank run, where a large number of bank clients decide to withdraw all their deposits at the same time. Consequently the bank is unable to fulfil contracts with other market participants or banks which leads to shortfalls in other sectors triggering a cascade of failures and a system-wide breakdown. In the last decade two major systemic risks lead to political and financial crisis, the global crisis in 2008 and the ongoing European debt crisis. The latter in combination with the inability of certain European states to place government bonds on the international market lead to the breakdown probability of the EURO-Zone.

In their study from 2010, Billio et al. [3] proposed that the two largest eigenvalues can be used to measure the underlying systemic risk of hedge funds, banks, brokers and insurance companies. In addition they used the Granger-Causality-Test [4] to illustrate the relation between these market sectors [3, 5]. These studies showed the increasing entanglement of the big four in financial markets. By this, they outlined a predictive indicator to systemic risk. The study also states that the banking and hedge fund sector seize dominant roles. This idea was advanced by Zheng et al. in 2012 by using principle component analysis on a correlation matrix consisting of 10 US sector indices. They showed that the steepest increase in the first four largest eigenvalues is connected to the systemic risk of the chosen indices. At the same time, the change in the four largest eigenvalues can be used as a precursor indicator. The magnitude of the increase translates to a higher interconnectedness of the 10 US sector indices and its risk aversion. [6, 7]

Former studies based their measure of systemic risk upon the relation between banks [8–13] and others focused more on time-series analysis with different risk-measures [9, 14–19]. The idea of news contagion is starting to become a part of systemic risk analysis [20–22]. We suggest that when studying systemic risks, one also has to take market participants behaviour, like banks, into account. The investors general goal is to generate return with a low risk exposure. The mean-variance model by Markowitz [23] was the first model capturing this need. Therefore we connect the ground-state portfolios spanning the efficient frontier to systemic risks [24]. A sharp change in the suggested portfolio is connected to the search of a new low overall risk. When all market participants are in that state the breakdown probability increases due to their increased connectivity [25]. The problem of selecting the optimal portfolio composition translates to the risks within the underlying dataset. An increase in the number of negative options correlates to a higher breakdown probability of the monitored dataset, since the number of stable configurations decreases. In this paper we introduce a measure called investing strategy *M*, which approximates the market participants behaviour by the ground-states of the mean-variance model with real world constraints. The time derivative of the average investing strategy peaks at the global financial crisis of 2008, the EURO-dept crisis and the Chinese stock market crash in 2015.

## Methods

### The mean-variance model

The mean-variance model was introduced by Markowitz in 1952 [23] and is one of the cornerstones of modern portfolio theory [26]. The objective is to maximise the average portfolio return

$$R_{P}=\sum_{i=1}^{N}r_{i}p_{i}$$ (1)

of portfolio weights *P* = (*p*_1_, *p*_2_, …, *p*_*N*_)^*T*^ with expected returns *r*_*i*_ for each asset *i* and minimise its variance

$$var_{P}=\sum_{i,j}p_{i}C_{ij}p_{j}$$ (2)

with the covariance matrix *C*_*ij*_. Both the covariance matrix and the expected returns are calculated from logarithmic returns

$$r_{i}\left(t\right)=\log\left(Price_{i}\left(t\right)\right)-\log\left(Price_{i}\left(t-1\right)\right)$$ (3)

where *t* denotes the time and runs through a single period Ω = {*t*|*T* − *ω* ≤ *t* ≤ *T*} with lag *ω* and the final datapoint at time *T*. The classical approach is constraining the portfolio to positive values *p*_*i*_ ≥ 0 ∀*i* and therefore preventing short-selling positions. Since an investor is bound by his available budget, all portfolio weights must sum up to one. This model quantifies the risk of a selected portfolio by using the variance and balances it with the expected return. Therefore an investor is able to choose a portfolio with the highest possible return at a certain risk level [27]. From an investors point of view, the classical model is not capturing real world demands of maintaining a portfolio. When constructing a portfolio, one has to account for transaction costs, which manifest in the cardinality of a portfolio. The three main constrictions a real investor has to consider [28], are the buy-in threshold *τ*, which defines a level of minimal investment in any asset *i*; the base unit of investment *ζ*; and the cardinality constraint *N*_*d*_, which sets the number of desired assets an investor wishes to manage. Introducing these real world demands to the model, it develops the portfolio selection problem from a quadratic programming problem to a NP-complete problem [29]. Due to the practical implications of portfolio managing, the cardinality constraint portfolio selection problem has been researched extensively [26, 28–39]. Under real market conditions an investor can profit from a decline in stock prices by purchasing short-options. Including this extension, every portfolio has to fulfil

$$\sum_{i}\left|p_{i}\right|=1$$ (4)

when cycling through Ω. This evolves the budget constraint to negative portfolio options, under the restriction not to invest over-proportionally in declining prices. The objective function

$$H=-\lambda R_{P}+\left(1-\lambda \right){\text{var}}_{P}$$ (5)

where *R*_*P*_ corresponds to the portfolio return and var_*P*_ to its variance, is minimised under the constraints of budget, cardinality and buy-in. In the context of portfolio selection, the parameter *λ* controls the level of risk one is willing to accept for a certain amount of return *R*_*P*_. This non trivial optimisation problem is solved by a tailored simulated annealing algorithm [40].

### The investing strategy

A general investing strategy *M* is based on the efficient frontier (EF), which is constructed by varying the parameter *λ* from 0 to 1 where for each *λ* an optimal or ground-state portfolio P* exists with a tuple (var_P*_,*R*_P*_) on the risk return plane. To quantify the investing strategy *M*, the sum of all $P_{i}^{*}$ is used, which is similar to a physical magnetisation [41] and weighted with a density function *ρ*(*λ*)

$$M_{\rho }\left(T,\omega \right)=C^{-1}\cdot \int_{0}^{1}d\lambda \;\rho \left(\lambda \right)\sum_{i=1}^{N}P_{i}^{*}\left(\lambda ,T,\omega \right)=C^{-1}\cdot \int_{0}^{1}d\lambda \cdot \rho \left(\lambda \right)\cdot m\left(\lambda ,T,\omega \right)$$ (6)

with $C=\int_{0}^{1}d\lambda \rho \left(\lambda \right)$ as a scaling constant. Therefore the investing strategy *M* indicates which proportion of portfolios along the efficient frontier, based upon the chosen density function *ρ*, should be invested into shorts or longs. The lagged time derivative

$$\partial_{t-l}M\left(T\right)=M\left(T\right)-M\left(T-l\right)$$ (7)

measures the changes in the investing strategy between two points in time *T* separated by *l* time-units.

A change in the behaviour of an average investor can be used as an early warning sign [42]. This idea is exploited by Eq (6). Since one does not know every market participant and their investments, the investing strategy approximates the investor ensemble by the ground-state P*(*λ*) for a certain risk-level. A shift in the ground-states along the EF leads to an overall changed market sentiment and therefore investors have to reevaluate their investments. In terms of a phase transition, a new basin is emerging and system participants, who are effectively sampling the market, will start to occupy that state [25, 42]. To move to the new state investors have to sell and buy new assets, which results in a higher trading volume, which in turn is connected to a higher volatility [43]. Assuming that investors are bound to the constraints introduced above, it is possible to detect such periods by monitoring the changes in the investing strategy in Eq (7)

### Data

All 37 US indices (Table 1) were downloaded by the QUANDL [44] interface. If for an observed time-frame Ω data is missing, the whole index is omitted for the analysis of the EF and its corresponding investing strategy. This is done in order to replicate the choices an investor has at a certain time with a given set of parameters.

**Table 1:** List of all US indices used to calculate the efficient frontier and their available time-frames. All Data was downloaded by the QUANDL interface [44]. (See S1 Table)

	First	Last
DJINDUSTRIAL	1900-01-31	2016-02-29
SP500	1950-01-31	2016-02-29
Morgan Stanley Cyclical Index	1978-01-31	2014-07-31
NYSE ARCA Major Market Index	1983-04-30	2016-02-29
NYSE AMEX Computer Technology Index	1983-08-31	2016-02-29
NYSE AMEX Oil Index	1983-08-31	2016-02-29
PHLX Gold/Silver Sector Index	1983-12-31	2016-02-29
NYSE ARCA Institutional Index	1986-10-31	2016-02-29
PHLX Utility Sector Index	1987-09-30	2016-02-29
NYSE AMEX Airline Index	1992-10-31	2016-02-29
NYSE AMEX Pharmaceutical Index	1994-02-28	2016-02-29
NYSE AMEX Natural Gas Index	1994-03-31	2016-02-29
Morgan Stanley Consumer Index	1994-03-31	2013-10-31
NYSE AMEX Securities Broker/Dealer Index	1994-04-30	2016-02-29
PHLX Semiconductor Index	1994-05-31	2016-02-29
CBOE S&P Healthcare Index	1994-12-31	2016-02-29
NYSE AMEX Biotechnology Index	1994-12-31	2016-02-29
MSCI US REIT Index	1995-06-30	2015-12-31
Interactive Week Internet Index	1995-10-31	2013-10-31
CBOE Gold Index	1996-04-30	2015-02-28
NYSE ARCA Networking Index	1996-07-31	2016-02-29
PHLX Oil Service Sector Index	1997-02-28	2016-02-29
PHLX Housing Sector Index	2002-07-31	2016-02-29
NASDAQ Bank (BANK)	2003-01-31	2016-02-29
NASDAQ Biotechnology (NBI)	2003-01-31	2016-02-29
NASDAQ Computer (IXCO)	2003-01-31	2016-02-29
NASDAQ Financial 100 (IXF)	2003-01-31	2016-02-29
NASDAQ100	2003-01-31	2016-02-29
NASDAQ Industrial (INDS)	2003-01-31	2016-02-29
NASDAQ Insurance (INSR)	2003-01-31	2016-02-29
NASDAQ Telecommunications (IXTC)	2003-01-31	2016-02-29
NASDAQ Transportation (TRAN)	2003-01-31	2016-02-29
NYSE TMT Index	2004-01-31	2016-02-29
SIG Oil Exploration & Production (EPX)	2005-12-31	2016-02-29
Wilshire US REIT Index	2009-04-30	2016-02-29
Wilshire US Real Estate Securities Index	2009-04-30	2016-02-29
TSX Global Gold Index (TTGD.TO)	2012-01-31	2016-02-29

## Results and Discussion

In order to show that *M* is a possible early warning sign, we calculate the logarithmic monthly returns of 37 US indices and solve the portfolio selection problem (PSP) under the constraints that the portfolio has to consist of *N*_*d*_ = 5 indices and the buy-in *τ* as well as the base unit of investment *ζ* is set to 10^−4^. The efficient frontier is constructed by *n* = 501 different *λ*’s equidistant distributed from 0 to 1. In order to capture the average over the efficient frontier, *ρ*(*λ*) is set to a constant value. We note that the given parameters are common values used in cardinality constraint optimisation problems, but we also tested different sets of parameters and density distributions *ρ* with similar results (see S1 Text).

**Fig 1:**
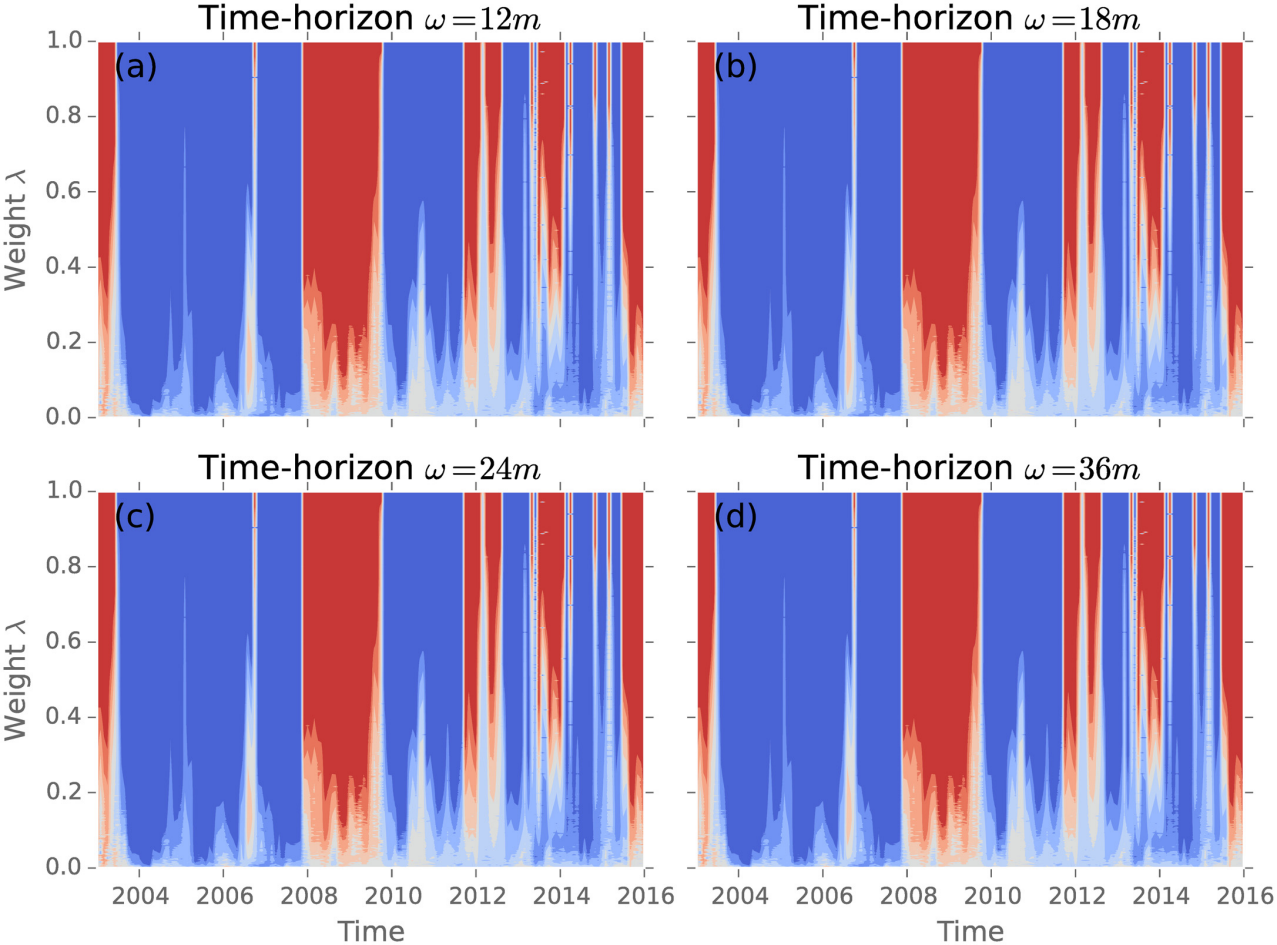
The investing strategies calculated for different time-horizons from January 2003 until January 2016. (a) *ω* = 1 year; (b) *ω* = 18 months; (c) *ω* = 2*y* and (d) *ω* = 3*y*; Blue signals a majority of positive portfolio weights, while red is related to dominantly negative weights in the solution. In all time-windows (a)-(d) the global financial crisis is depicted with negative weights.

Fig 1 shows the time-development of the suggested investment strategies *m*(*t*, *λ*) starting in 2003. These are deduced from the mean-variance model with different time-horizons *ω*. Blue colours represent stable phases with mostly positive weights, while red colours indicate phases with high systemic risk, due to a large negative weight proportion. In all depicted time-horizons, the global financial crisis is present from 2007 until 2009. The longer the time-horizons, the longer it takes to recover to stable conditions. Optimal portfolios are dependent on the time-horizon, since a different time-horizon *ω* results in a divers set of returns [28].

**Fig 2:**
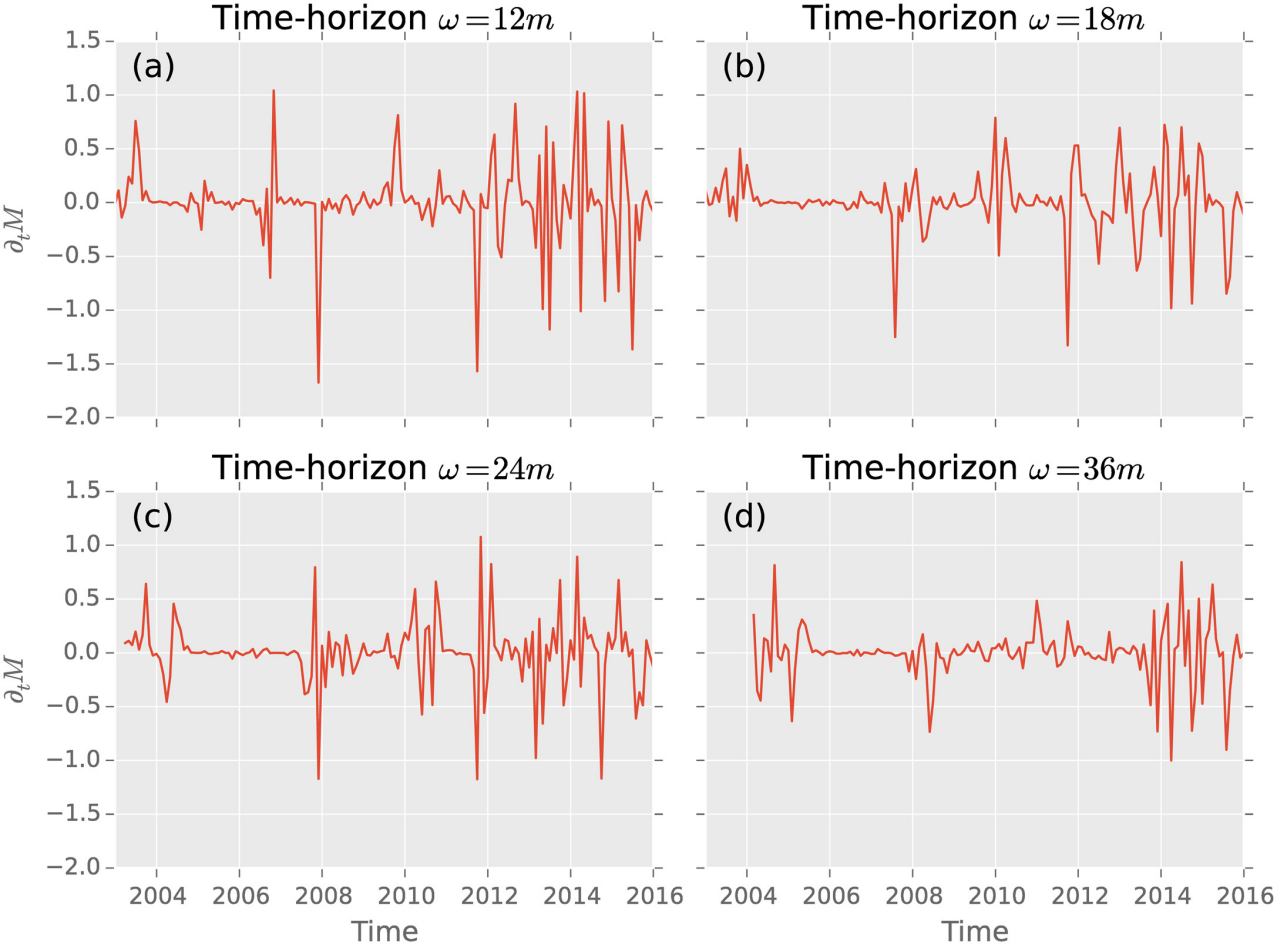
(a)–(d) depict the time derivation of the average investing strategies for different time-horizons. In (a) with *ω* = 1 year the three major crisis in the time from 2003 until 2016 exhibit the three largest drops. On longer time-horizons (b) *ω* = 18 months, (c) *ω* = 2 years and (d) *ω* = 3 years many signals mix and the decreases are not highly developed. Especially since 2013 volatility clustering is observed.

We show dependence on time in Fig 2, starting with a time-horizon of 12-month up to 36-month. These time-horizons are usually used in literature [3, 6, 14, 30]. For the purpose of this paper, we chose our observation time-horizon to be *ω* = 12months, since it exhibits the most distinctive peaks. Choosing a larger *ω* leads to a smoothing of the signal, since shocks happen on a shorter time-scales and loose their impact on the statistics over longer times. [6]

**Fig 3:**
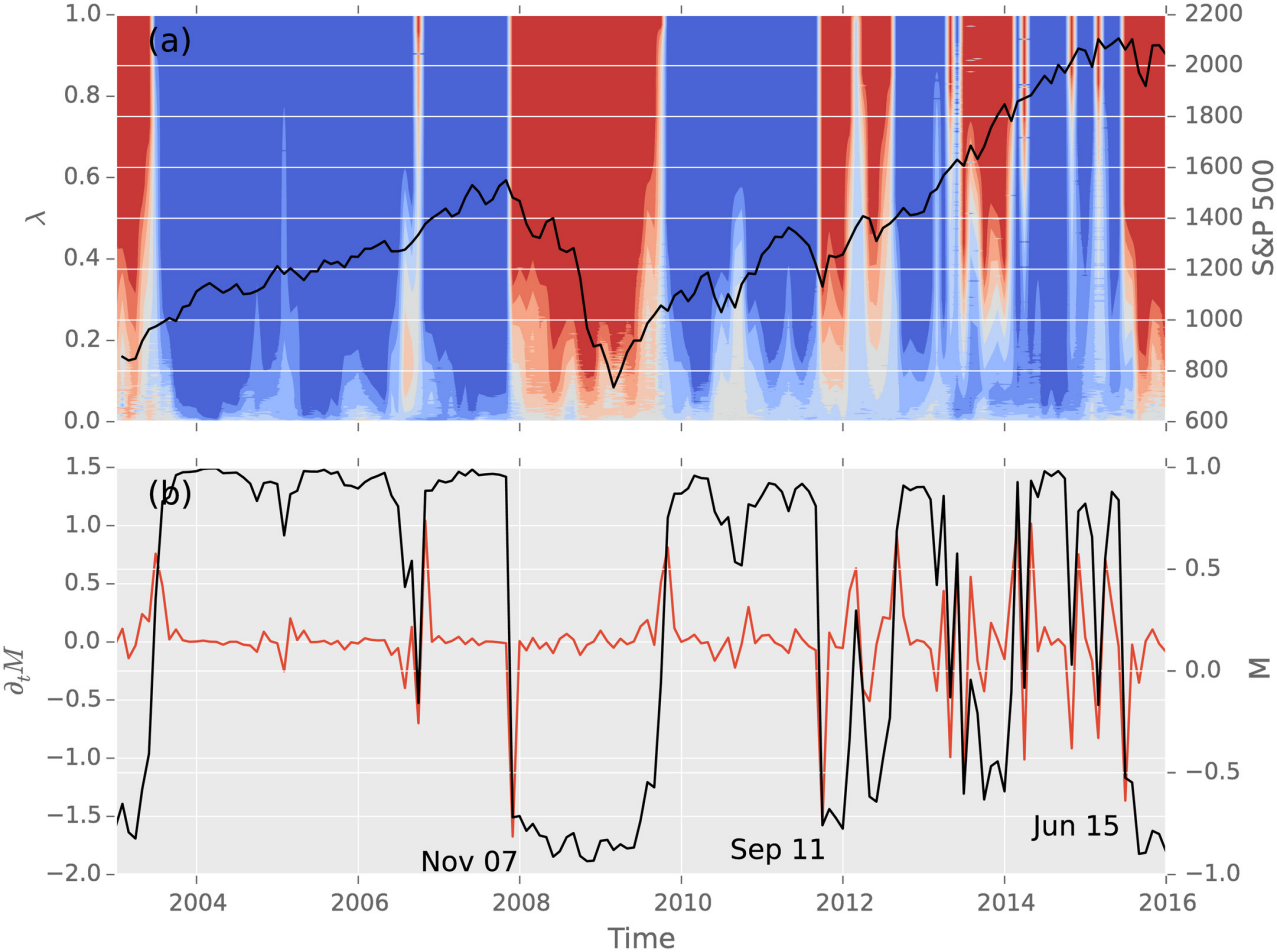
Comparison of the average investing strategy and its recordings with the S&P 500 index, in the *ω* = 12 months time-horizon. (a) shows the heat-map with varying risk level *λ* on the left y-axis and the logarithmic values of the S&P 500 index on the right y-axis. (b) shows on the left the changes in the average investing strategy (right y-axis). The slumps in November 2007, September 2011 and June 2015 fall in the times of sub-prime/financial crisis, EURO-Zone debt crisis and Chinese stock market crash.

In November 2007, the average investing strategy *M*(*t*) dropped from 95% to −73% (see Fig 3). This decrease by ∂_*t*_ *M* = −168% is the largest drop in the 12 month time-horizon as well as in the observation time starting in 2003. Afterwards *M* kept decreasing to the minimum of *M* = −94%, after recovering in October 2009. During this time of financial turmoils, the average investing strategy was ranging between −57% and −94%. In September 2011 a slump of ∂_*t*_ *M* = −157% occurred. This corresponds to the time where speculation about the survival of the EURO-Zone took place. In this crisis the average investing strategy never drops below −77%. The next largest decline is observed in June 2015 with ∂_*t*_ *M* = −136%. Over the following months the average investing strategy was slipping up to *M* = −90% in January 2016. During this timeframe, the Chinese market was suffering from turmoils. A drop to these values has not been seen since the financial crisis.

**Fig 4:**
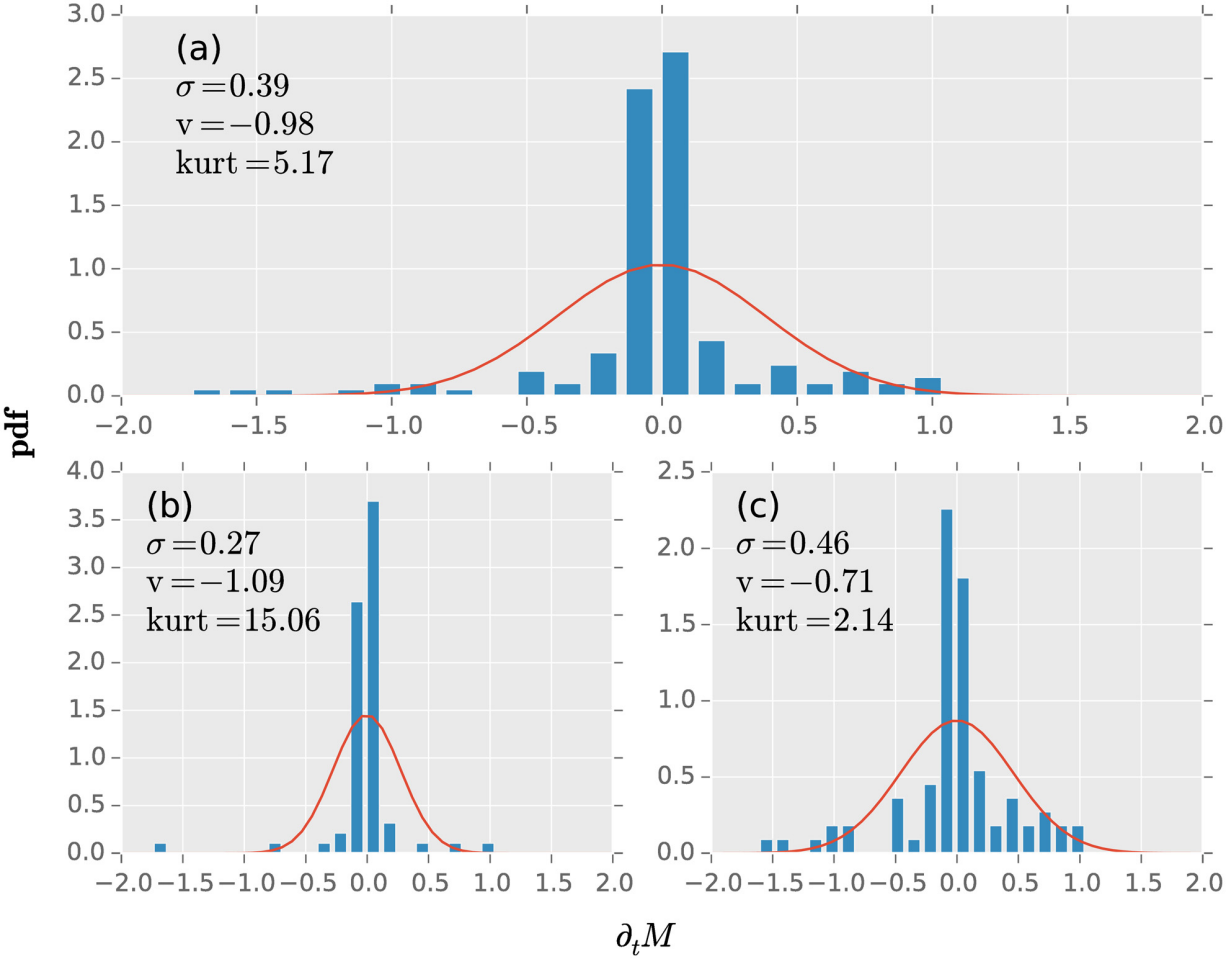
The average investing strategy time derivation probability functions (pdf) of (a) the whole observation time from 2003 until 2016; (b) from 2003 until 2009; and (c) from 2009 until 2016. Between 2003 and 2009 the pdf densely centered around 〈∂_*t*_ *M*〉 = −0.00 ± 0.04 exhibiting a kurt = 15.06 ± 1.07 and an extreme event at −1.5. Afterwards the 〈∂_*t*_ *M*〉 = −0.00 ± 0.05 is also centered. The kurtosis dropped to kurt = 2.14 ± 1.07 and shows a standard deviation of *σ* = 0.46 ± 0.05 in comparison to *σ* = 0.27 ± 0.08. The skewness changed from v = −1.09 ± 2.44 before 2009 and v = −0.71 ± 0.36. The whole observation time pdf has 〈∂_*t*_ *M*〉 = −0.00 ± 0.03 with *σ* = 0.39 ± 0.04, kurt = 5.17 ± 1.60 and v = −0.98 ± 0.50. Note, that these values were estimated with 1000 bootstrapping samples. The comparison plot is a normal distribution with zero mean and the corresponding standard deviations for each time frame.

Next we study the behaviour of the changes in average investment strategy on a twelve month time-horizon before and after the financial crisis (Fig 4). We select the up-move from unstable to stable in October 2009 as the separating point of the two studied time-periods. The overall probability density function (Fig 4a) from 2003 to 2016 is skewed with v = −0.98. In comparison with the normal distribution with the same standard deviation *σ* = 0.39 a non vanishing probability of extreme events exit. We suggest, that these fat tails are related to the systemic risk.

Separating the time-series into two parts, before (Fig 4b) and after October 2009 (Fig 4c), we observe a difference in the kurtosis from kurt = 15.06 to kurt = 2.14. During the time before October 2009, the distribution is dominated by the outliers around the mean 〈∂_*t*_ *M*〉 ≈ − 0.00. While the skewness remains with v = −1.09 before and v = −0.71 after in the same range, the standard deviation almost doubles from *σ* = 0.27 to *σ* = 0.46.

**Fig 5:**
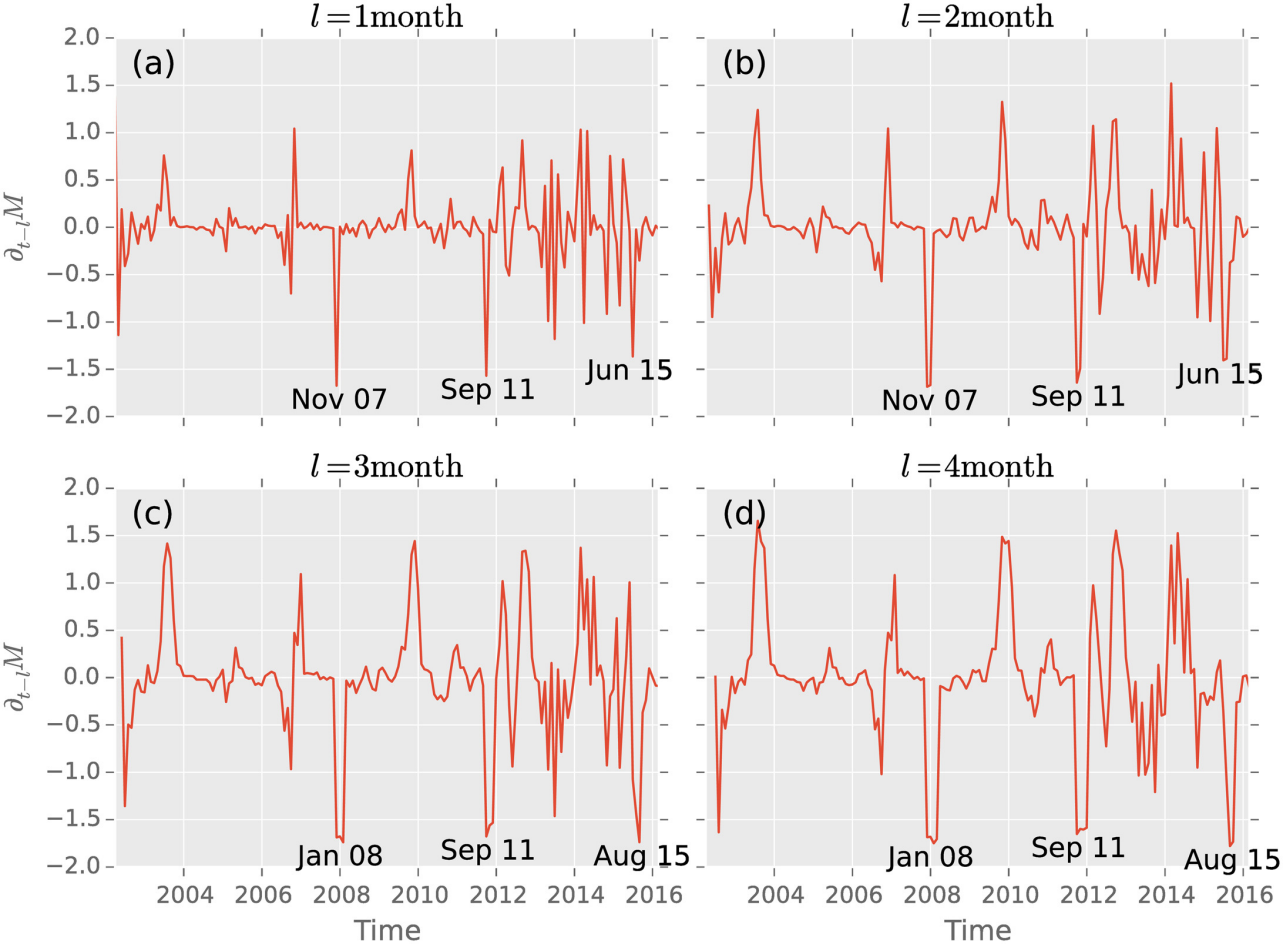
The three largest drops in time derivation with lag (a) *l* = 1 month, (b) *l* = 1 month, (c) *l* = 3 month and (d) *l* = 4 month of the average investing strategy in the time-horizon *ω* = 12months. With lag *l* = 1, 2 these occur ordered by strength November 2007, September 2011 and June 20015. Lag *l* = 3, 4 this changes to August 2015, January 2008 and September 2011.

In order to quantify the highest peak, Fig 5 shows a time-derivative with different lags defined by

$$\partial_{t-l}M=M\left(t\right)-M\left(t-l\right).$$ (8)

The sharpest change occurs in the derivative with *l* = 1 month in October 2007, which also marks the highest peak in the S&P 500 in its history. Increasing the lag *l* to higher values the peak broadens since after a drop the magnitude can increase. We find that in Fig 5a and 5b the absolute peak occurs in November 2007, while with longer lags *l* = 3, 4 months in Fig 5c and 5d the peak is found in January 2008. Similarly the last large decrease is found in June 2015 (Fig 5a and 5b) or in August 2015 (Fig 5a and 5b).

Due to globalisation processes, financial markets have become more entangled in the last 25 years. Thus the cross-correlation between markets and sectors increased [45–47], which makes the sectors more suggestible to events in others. This cascading is connected to systemic risks. This paper proposes that systemic risk can be quantified by the behaviour of the average investment strategy of a portfolio selection problem. Abrupt changes signal that the stable configuration has to adapt to changing market situations. Since every investor has to balance return with risk, one can track these financial reflections ahead of time. We showed that the 12 month window of monthly log returns is a suitable parameter range. Since the optimal solutions found translate to the most efficient way of investing, according to Markowitz, they trace the general market behaviour. Therefore our indicator can be used on large data sets with many different economic sectors and needs no selection process before hand. Fig 3 displays the investing strategies, its average and time-derivative and compares it to the log values of the S&P 500. Besides the negative phase between November 2007 and October 2009, a new phase has formed in June of 2015. The latter may be connected to the Chinese stock market crash. The evaluated time-frame shows that the three major crisis, which took place are the three greatest decreases in the average investing strategy.

The fluctuations present between October 2009 and June 2015, are in our opinion due to high uncertainties. It is not possible to settle into a phase for a long time period. In analogy to physics, this is present when a magnet is at critical temperature. Despite the high fluctuations, the absolute value of the average investing strategy does not drop below *M* = −60%, while in the financial crisis this value was well undershot.

In conclusion, we propose that sharp changes in the investing strategies, relying on the efficient frontier by Markowitz, serve as an indicator for systemic risks which can cascade to all market sectors of an economy.

## Supporting Information

S1 Text Effect of *ρ* on the AIS with a comparison to an eigenvalue analysis. (PDF)

S1 Table List of QUANDL Codes used. (CSV)
