# Supplementary material for: Quantifying Systemic Risk by Solutions of the Mean-Variance Risk Model
Source: PLoS One. 2016 Jun 28;11(6):e0158444. doi: 10.1371/journal.pone.0158444 (PMC4924827; doi:10.1371/journal.pone.0158444)
Supplement: S1 Text — (PDF) [file pone.0158444.s001.pdf]

# Supplementary: Quantifying systemic risk by mean-variance portfolio ground-states

JJ, AE, IM

May 24, 2016

In the article a uniform distribution of investors along the efficient frontier was assumed, in order to capture an average over the a general market. Yet, it is possible that $\rho(\lambda)$ takes other forms. We study the case of $\rho$ being a $\delta$-function around a certain value in Fig. (1). This would be the case when every investor is only interested in one risk-level $\lambda$. We tested for $\lambda = 0\%, 1\%, 10, \%, 50\%, 100\%$. In general even one certain risk-level exhibits the distinctive peak when reweighing the portfolio takes place. It is also possible, that most of the investors are packed around a certain $\lambda$. In order to mimic that, we show in Fig. (2) the results of choosing $\rho$ as a normal distribution centered around $\mu_\lambda = 0\%, 10\%, 25\%, 50\%, 100\%$ and a standard deviation of $\sigma_\lambda = 1/8$. Choosing a broader distribution leads to values similar as in the uniform case.

As our parameters we set $\omega$ to be 12 months, with $\tau$ and $\zeta$ equal to $10^{-4}$ and restricting the investors to $N_d = 5$ US indcies.

As a last step, we compare in Fig. (3) our early warning indicator to the indicator proposed by Zheng on our dataset. The sum of normalised eigenvalues $\alpha_i$ is

$$\Lambda_k = \sum_{i=0}^{k} \alpha_i \tag{1}$$

where the normed eigenvalues $\alpha_i$ are sorted in decreasing order. In order to capture between 85% and 95% of the market, we set $k = 5$ and plot the changes in $\Lambda_5$ normalised by the absolute maximum. The same was done with $M$. Comparing both indicators reveals, that the eigenvalues and the average investing strategy signal November 2007 as a critical date.

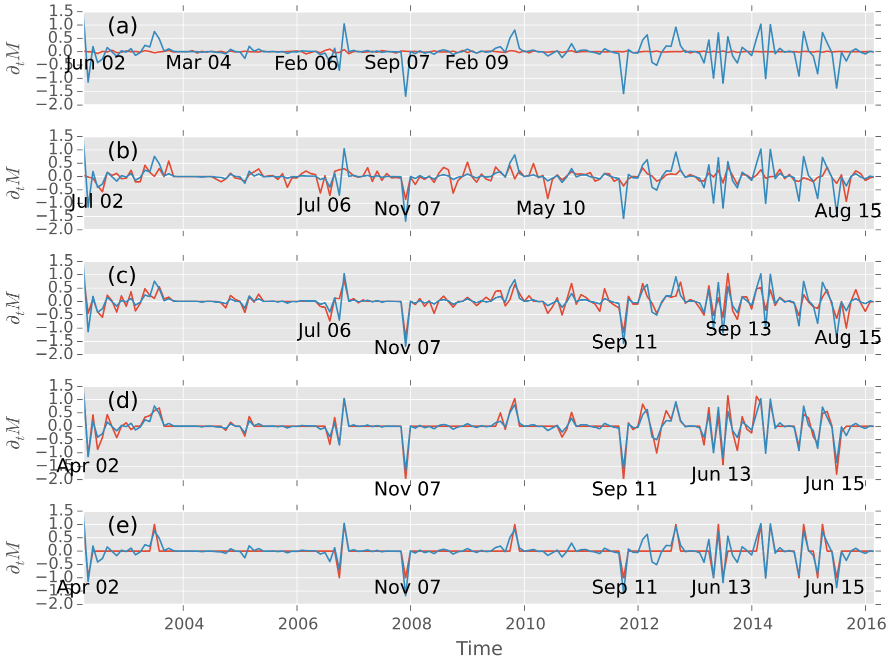


Figure 1: Time derivative of the investing strategy with $\rho(\lambda) = \delta(\lambda - k)$ for different risk-levels: (a) $\lambda = 0\%$ (b) $\lambda = 1\%$ (c) $\lambda = 10\%$ (d) $\lambda = 50\%$ (e) $\lambda = 100\%$. In all risk-levels the distinct peak in November 2007 is present as well as the higher volatility after 2009.

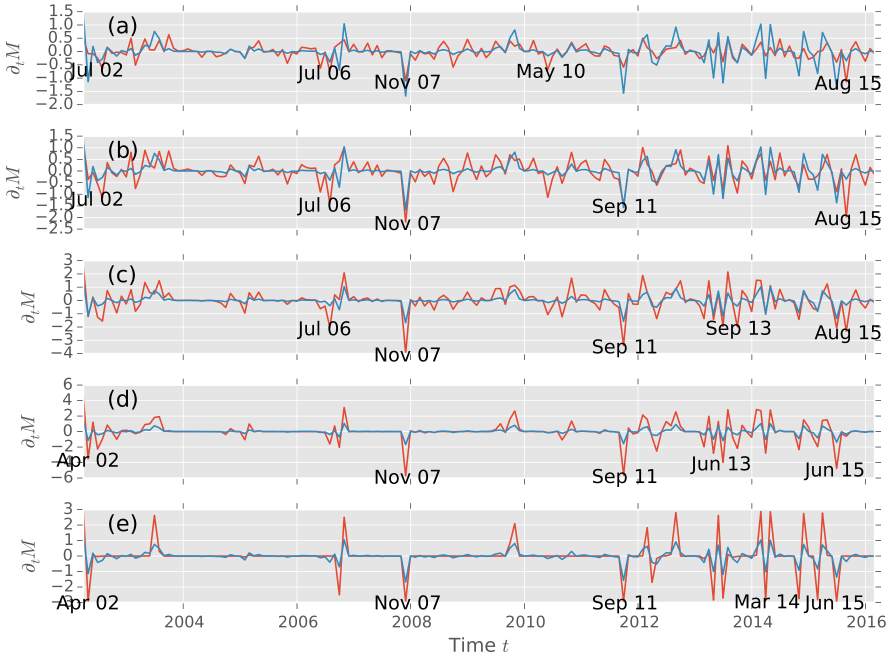


Figure 2: The time derivative of $M$ with a normal distribution centered around different $\lambda$'s with a scaling factor of $\sigma_\lambda = 1/8$. In (a) only very risk aware investors are considered by setting $\mu_\lambda = 0\%$; form (b) with $\mu_\lambda = 10\%$; (c) $\mu_\lambda = 25\%$; (d) $\mu_\lambda = 50\%$ to (e) with $\mu_\lambda = 100\%$ one can read the points in time where reshuffling is about to take place. Increasing $\sigma_\lambda$ leads to similar distributions as a uniform density function.

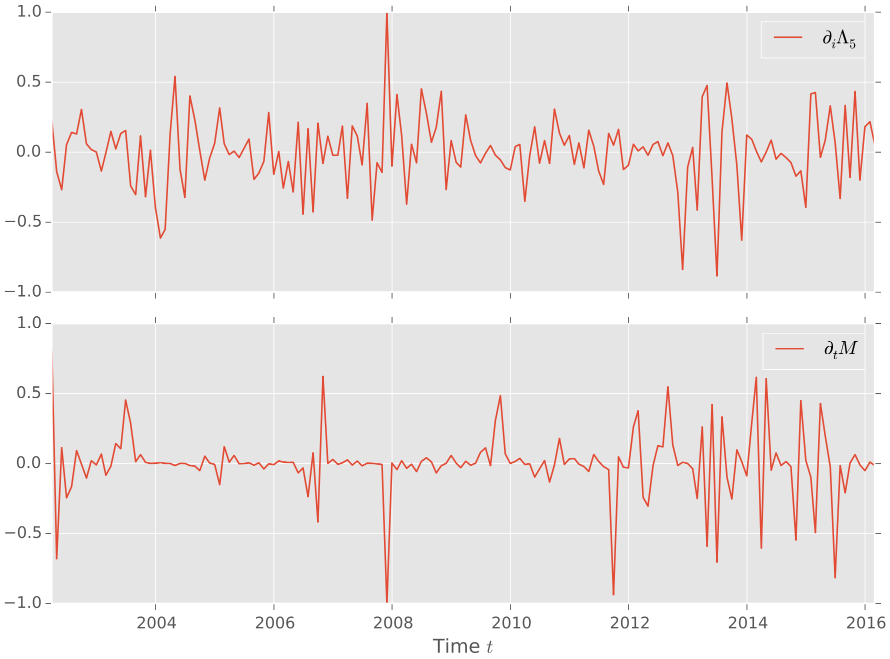


Figure 3: Comparison between the changes in the 5 largest eigenvalues (upper) with the average investing strategy (lower). The strongest increase in coupling happened in November 2007. This coincides with the steepest decrease of $M$.In order to explain between 85% and 95% of the data, the five greatest eigenvalues were chosen to calculate the changes in the cross-correlation matrix.
